# Influence of Vincristine, Clinically Used in Cancer Therapy and Immune Thrombocytopenia, on the Function of Human Platelets

**DOI:** 10.3390/molecules26175340

**Published:** 2021-09-02

**Authors:** Li-Ming Lien, Wan-Jung Lu, Kuan-Hung Lin, Ling-Hsuan Kang, Ting-Yu Chen, Bo-Jung Lin, Yung-Chang Lu, Chun-Yao Huang, Chun-Ming Shih, Hsuan Chen, Yao-Chou Tsai, Ray-Jade Chen, Joen-Rong Sheu

**Affiliations:** 1 Department of Neurology, Shin Kong Wu Ho-Su Memorial Hospital, Taipei 111, Taiwan; M002177@ms.skh.org.tw; 2 Department of Neurology, School of Medicine, College of Medicine, Taipei Medical University, Taipei 110, Taiwan; 3 Department of Medical Research and Traditional Herbal Medicine Research Center, Taipei Medical University Hospital, Taipei 110, Taiwan; luwj@tmu.edu.tw; 4 Department of Pharmacology, School of Medicine, College of Medicine, Taipei Medical University, Taipei 110, Taiwan; linkh@mmc.edu.tw (K.-H.L.); y0513260323@tmu.edu.tw (T.-Y.C.); spring38012@gmail.com (B.-J.L.); 5 Graduate Institute of Metabolism and Obesity Sciences, College of Nutrition, Taipei Medical University, Taipei 110, Taiwan; 6 Institute of Biomedical Sciences, MacKay Medical College, New Taipei City 252, Taiwan; 7 Graduate Institute of Medical Sciences, College of Medicine, Taipei Medical University, Taipei 110, Taiwan; m120107024@tmu.edu.tw; 8 Department of Surgery, School of Medicine, College of Medicine, Taipei Medical University, Taipei 110, Taiwan; 9 Department of Medicine, MacKay Medical College, New Taipei City 252, Taiwan; yungchanglu@hotmail.com; 10 Department of Orthopaedic Surgery, MacKay Memorial Hospital, Taipei 104, Taiwan; 11 Department of Medical Research, MacKay Memorial Hospital, Taipei 104, Taiwan; 12 Division of Cardiology, Department of Internal Medicine, School of Medicine, College of Medicine, Taipei Medical University, Taipei 110, Taiwan; cyhuang@tmu.edu.tw (C.-Y.H.); cmshih53@tmu.edu.tw (C.-M.S.); shirlleychan@gmail.com (H.C.); 13 Taipei Heart Institute, Taipei Medical University, Taipei 110, Taiwan; 14 Division of Cardiology and Cardiovascular Research Center, Department of Internal Medicine, Taipei Medical University Hospital, Taipei 110, Taiwan; 15 Department of Urology, School of Medicine, College of Medicine, Taipei Medical University, Taipei 110, Taiwan; tsai1970523@tmu.edu.tw; 16 Department of Urology, Taipei Medical University Hospital, Taipei 110, Taiwan; 17 Division of General Surgery, Department of Surgery, Taipei Medical University Hospital, Taipei 110, Taiwan

**Keywords:** collagen, glycoprotein VI signaling, platelet activation, vincristine, immune thrombocytopenia

## Abstract

Vincristine is a clinically used antimicrotubule drug for treating patients with lymphoma. Due to its property of increasing platelet counts, vincristine is also used to treat patients with immune thrombocytopenia. Moreover, antiplatelet agents were reported to be beneficial in thrombotic thrombocytopenic purpura (TTP). Therefore, we investigated the detailed mechanisms underlying the antiplatelet effect of vincristine. Our results revealed that vincristine inhibited platelet aggregation induced by collagen, but not by thrombin, arachidonic acid, and the thromboxane A_2_ analog U46619, suggesting that vincristine exerts higher inhibitory effects on collagen-mediated platelet aggregation. Vincristine also reduced collagen-mediated platelet granule release and calcium mobilization. In addition, vincristine inhibited glycoprotein VI (GPVI) signaling, including Syk, phospholipase Cγ2, protein kinase C, Akt, and mitogen-activated protein kinases. In addition, the in vitro PFA-100 assay revealed that vincristine did not prolong the closure time, and the in vivo study tail bleeding assay showed that vincristine did not prolong the tail bleeding time; both findings suggested that vincristine may not affect normal hemostasis. In conclusion, we demonstrated that vincristine exerts antiplatelet effects at least in part through the suppression of GPVI signaling. Moreover, this property of antiplatelet activity of vincristine may provide additional benefits in the treatment of TTP.

## 1. Introduction

Platelet activation is involved in normal hemostasis [1,2]. When blood vessels are injured, circulating platelets will adhere to and activate the exposed extracellular matrix proteins (e.g., von Willebrand factor and collagen). The exposed collagen can induce platelet activation through glycoprotein VI (GPVI). Collagen binding to GPVI results in tyrosine kinase Syk recruitment and activation [3,4]. A signaling complex in turn forms and triggers a signaling cascade that activates phospholipase Cγ2 (PLCγ2) and its downstream effectors, including protein kinase C (PKC) and mitogen-activated protein kinases (MAPKs) [3,4,5], leading to platelet activation and further recruitment of more platelets from the blood. In addition, integrin α2β1, another collagen receptor, can undergo platelet activation-dependent conformational changes to efficiently bind to collagen that further strengthens adhesion [6,7,8]. Platelets finally form a firm platelet plug at the injury site to stop blood loss; however, under pathological conditions, they are prone to uncontrolled activation and aggregation and may cause vessel occlusion.

**Figure 1:**
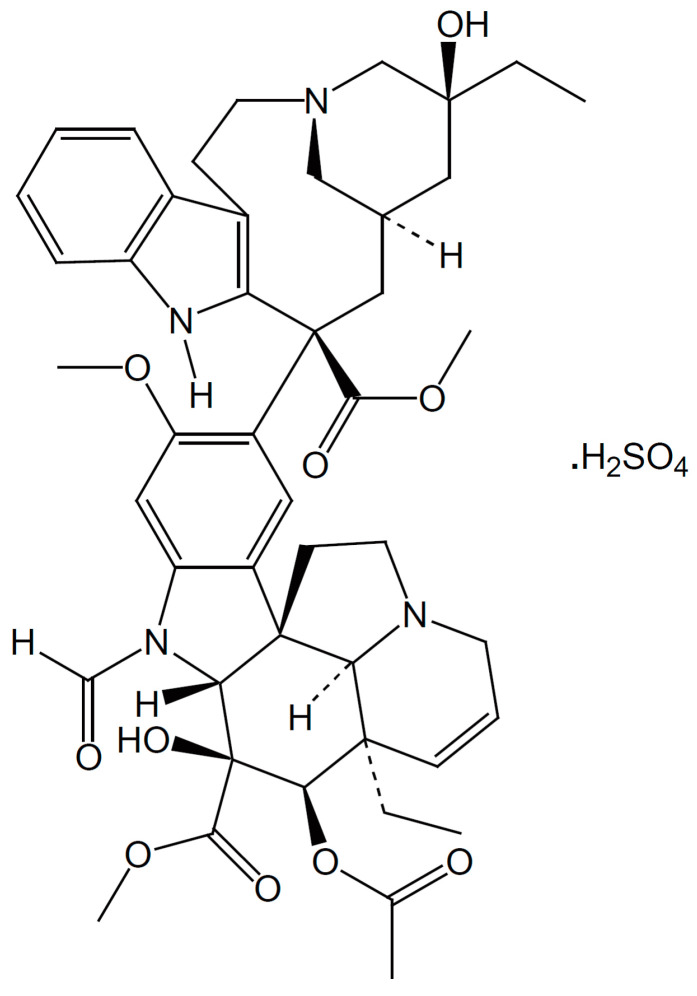
The structure of vincristine.

Vincristine, a naturally occurring vinca alkaloid (Figure 1), is present in the leaves of the plant *Catharanthus roseus* [9,10]. Vincristine is an antimicrotubule agent used for treating childhood and adult malignancies, such as acute lymphoblastic leukemia (ALL) and Hodgkin disease. Its antitumor effects may be mediated by causing cell arrest in the metaphase and cell apoptosis. Vincristine has better responses in hematological malignancies than in solid tumors [9,10]. In addition, vincristine or vincristine-loaded platelets are used to treat refractory immune thrombocytopenia (ITP) [11,12,13,14]. It has been reported to be an effective approach for transplantation-associated thrombotic microangiopathy [15].

Several studies have reported vincristine’s effects on platelet function in humans and animals. Steinherz et al. reported platelet dysfunction in vincristine-treated patients, whose platelets showed deficiency of secondary aggregation in response to various agonists [16]. White et al. also reported that vincristine did remove the microtubules of platelets and inhibited human platelet aggregation induced by various agonists, but complete prevention of microtubule dissociation had no corrective influence on vincristine-induced inhibition of platelet function, indicating that other mechanisms may be involved in this effect [17]. Moreover, controversial results were reported in dogs, in which the effect of vincristine on platelet function was evaluated [18,19,20]. These observations suggest that the effect of vincristine on human platelet function remains unclear. Moreover, antiplatelet agents were reported to be beneficial in thrombotic thrombocytopenic purpura [21].

Therefore, in the present study, we used washed human platelets of healthy subjects to determine possible mechanisms through which vincristine regulates platelet function.

## 2. Results

### 2.1. Vincristine Reduced Collagen-Induced Human Platelet Aggregation

**Figure 2:**
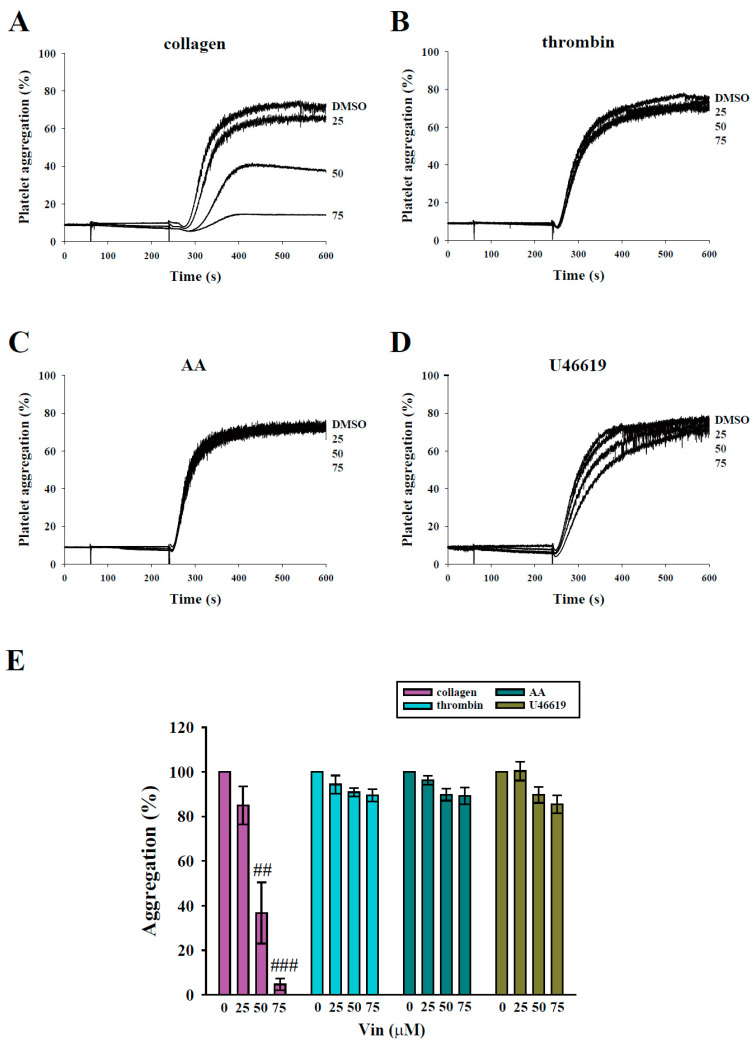
Effects of vincristine on platelet aggregation stimulated by various agonists in human platelets. Washed platelets (3.6 × 10^8^ cells/mL) were treated with vincristine (25–75 μM) or DMSO before the addition of 1 μg/mL collagen (**A**), 0.02 U/mL thrombin (**B**), 60 μM AA (**C**), and 1 μM U46619 (**D**). Panels (**A**–**D**) indicate the platelet aggregation curve, and panel (**E**) displays the statistical analysis of platelet aggregation (percent). Data in (**E**) are presented as mean ± standard error of the mean (*n* = 3). ^##^ *P* < 0.01 and ^###^ *P* < 0.001 compared with the DMSO (solvent control) group.

In the present study, we evaluated the influence of vincristine on human washed platelet aggregation induced by various agonists, including collagen, thrombin, AA, and U46619. The data revealed that vincristine (50 and 75 μM) markedly inhibited platelet aggregation induced by collagen (Figure 2A), but not by thrombin, AA, and U46619 (Figure 2B–D), indicating that vincristine exerts higher inhibitory effects on collagen-induced platelet activation. The results of statistical analysis are presented in Figure 2E. The IC_50_ value was approximately 40 μM. Thus, in the following experiments, two concentrations (40 and 75 μM) of vincristine were used.

### 2.2. Vincristine Inhibited Collagen-Induced Granule Release and Calcium Mobilization of Human Platelets

**Figure 3:**
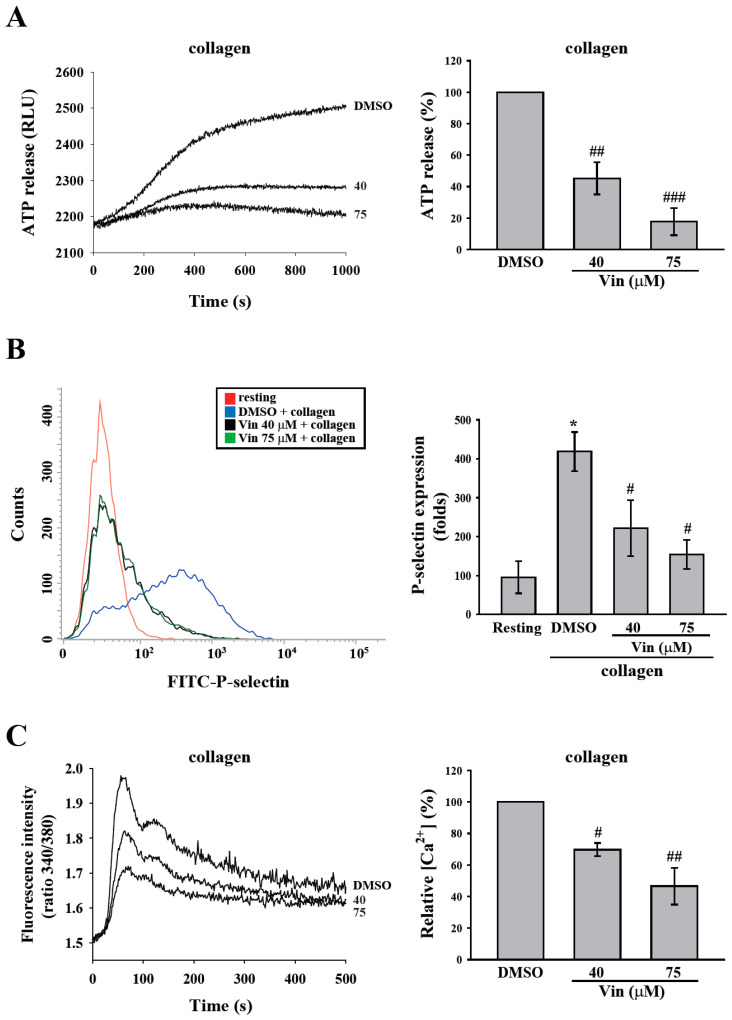
Effects of vincristine on collagen-induced granule release and calcium mobilization in human platelets. Washed platelets (3.6 × 10^8^ cells/mL) were treated with vincristine (40 and 75 μM) or DMSO and then stimulated with collagen (1 μg/mL) to trigger the release of ATP (**A**), the surface expression of P-selectin (**B**), and calcium mobilization, which were detected using luciferase/luciferin, FITC-P-selectin antibodies, and Fura-2, respectively. Data in (**A**–**C**) are presented as means ± standard error of the mean (*n* = 3). * *P* < 0.05 compared with the resting group. ^#^ *P* < 0.05, ^##^ *P* < 0.01, and ^###^ *P* < 0.001 compared with the DMSO (solvent control) group.

Platelet granule release and calcium mobilization are usually used to evaluate platelet activation. Thus, we determined the effect of vincristine on platelet function by measuring granule release and calcium mobilization. First, the release of dense granules and α-granules was measured by detecting ATP release and surface P-selectin expression. As shown in Figure 3A,B, vincristine attenuated ATP release and P-selectin secretion of human platelets in a dose-dependent manner in response to collagen. In addition, we measured calcium release through detection using Fura-2. The data showed a dose-dependent inhibition of calcium mobilization (Figure 3C). These findings revealed that vincristine inhibited collagen-induced platelet activation.

### 2.3. Vincristine Blocked Collagen-Induced Activation Signaling in Human Platelets

**Figure 4:**
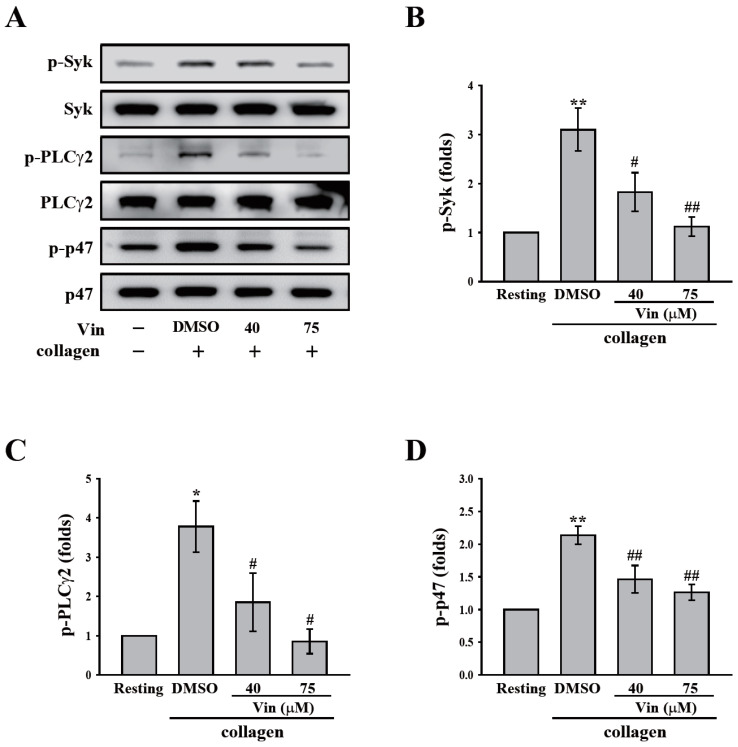
Effects of vincristine on collagen-mediated phosphorylation of Syk, PLCγ2, and the PKC substrate (p47) in human platelets. (**A**–**C**) Washed platelets (3.6 × 10^8^ cells/mL) were treated with vincristine (40 and 75 μM) or DMSO before collagen treatment (1 μg/mL). Protein extracts of platelets were subjected to Western blotting. The total and phosphorylated Syk, PLCγ2, and p47 were detected using specific antibodies. Profiles in (**A**) are representative examples of three similar experiments. Data in (**B**–**D**) are presented as the mean ± standard error of the mean (*n* = 3). * *P* < 0.05 and ** *P* < 0.01 compared with the resting group. ^#^ *P* < 0.05 and ^##^ *P* < 0.01 compared with the DMSO (solvent control) group.

**Figure 5:**
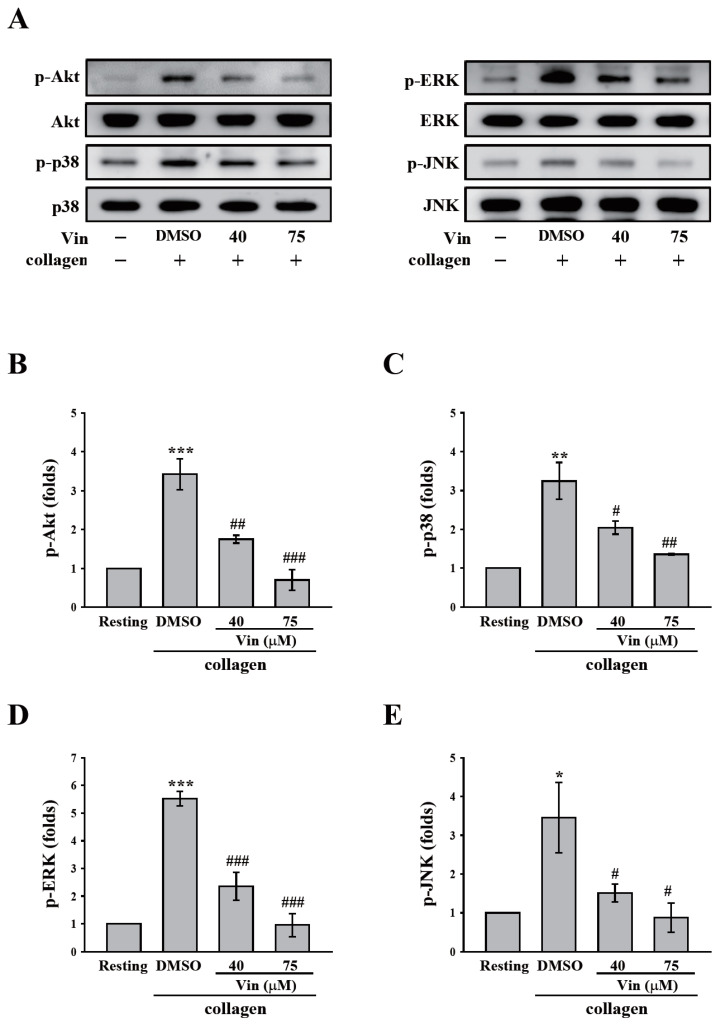
Effects of vincristine on collagen-mediated phosphorylation of Akt and MAPKs in human platelets. Washed platelets (3.6 × 10^8^ cells/mL) were treated with vincristine (40 and 75 μM) or DMSO before collagen treatment (1 μg/mL). Protein extracts of platelets were subjected to Western blotting. The total and phosphorylated Akt, p38 MAPK, ERK, and JNK were detected using specific antibodies. Profiles in (**A**) are representative examples of three similar experiments. Data in (**B**–**E**) are presented as means ± standard error of the mean (*n* = 3). * *P* < 0.05, ** *P* < 0.01, and *** *P* < 0.001 compared with the resting group. ^#^ *P* < 0.05, ^##^ *P* < 0.01, and ^###^ *P* < 0.001 compared with the DMSO (solvent control) group.

GPVI signaling is crucial for platelet activation induced by collagen. Thus, we further observed the effect of vincristine collagen-mediated GPVI signaling. The critical downstream regulators of GPVI, such as Syk and PLCγ2-PKC, were first determined in this study. The data revealed that vincristine (40 and 75 μM) inhibited the phosphorylation of Syk, PLCγ2, and the 47 kD PKC substrate (p47) (Figure 4A). The results of these individual statistical analyses are shown in Figure 4B–D. In addition, several proteins involved in collagen-mediated activation signaling, including Akt and MAPKs (p38 MAPK, ERK, and JNK), were observed. Similarly, vincristine inhibited the phosphorylation of Akt and MAPKs in a dose-dependent manner (Figure 5). Altogether, these findings demonstrated that vincristine can inhibit collagen-induced platelet activation in human platelets.

### 2.4. Vincristine Did Not Affect In Vitro and In Vivo Hemostasis

**Figure 6:**
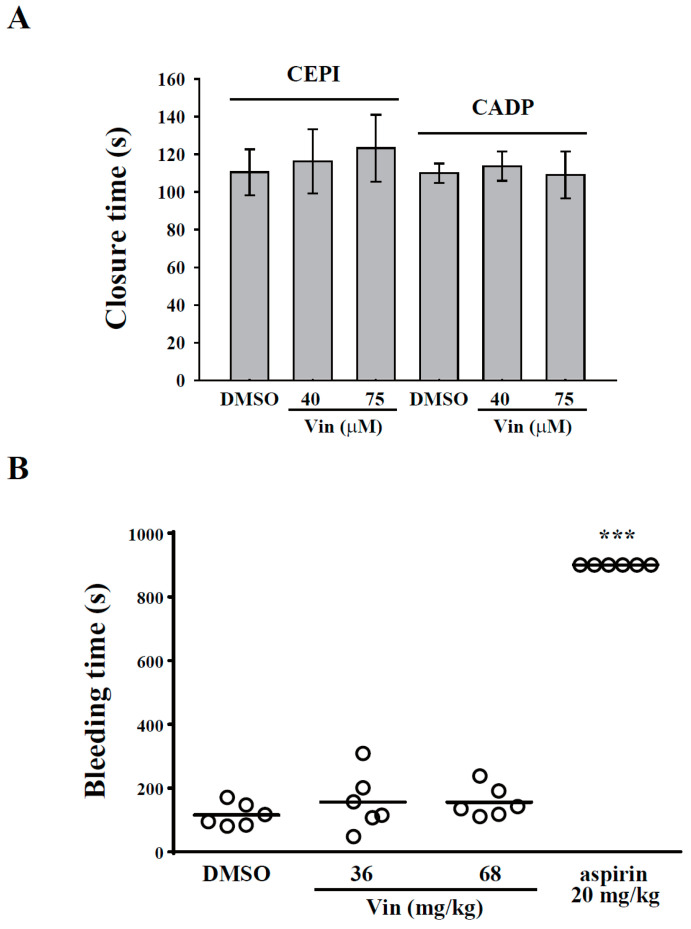
Effects of vincristine on in vitro and in vivo hemostasis. (**A**) Whole blood sample was collected in collagen/epinephrine or collagen/ADP and treated with DMSO and vincristine (40 and 70 μM). Closure time was then measured by platelet function analyzer-100 assay. (**B**) Mice were intravenously administered DMSO (solvent control), vincristine (36 and 68 mg/kg), or aspirin (20 mg/kg, positive control) for 10 min. Tail bleeding was induced by cutting the tail, and the bleeding time was recorded until the time when no sign of bleeding was observed for at least 10 s. Each point in the plot indicates one mouse (*n* = 6). Data in (**B**) are presented as the mean ± standard error of the mean (*n* = 6). *** *P* < 0.001 compared with the DMSO group.

In the present study, we examined whether vincristine affects hemostasis. PFA-100 analysis (in vitro) and a tail bleeding assay (in vivo) were performed. The PFA-100 system simulates the injured vessel with exposed collagen under a high shear rate. In this study, fresh human whole blood with DMSO or vincristine (40 and 75 μM) was placed in the cartridge coated with collagen/epinephrine (CEPI) or collagen/ADP (CADP), and the closure time was measured by the PFA-100 analyzer. The data revealed that the closure times of the DMSO group in CEPI and CADP cartridges were 110.5 ± 12.2 s and 110.0 ± 5.1 s, respectively (Figure 6A). However, vincristine showed no significant change compared with the DMSO group, indicating that vincristine did not affect hemostasis. For the tail bleeding assay in mice, the bleeding time, which was recorded after the tail was severed, in the DMSO group was 115.8 ± 14.9 s. In the aspirin group (positive control), the bleeding time of all the mice was >15 min (900 s) (Figure 6B). However, vincristine (36 mg/kg and 68 mg/kg) showed no significant change compared with the DMSO group. These findings further confirmed that vincristine did not affect hemostasis.

## 3. Discussion

In the present study, we demonstrated that the clinically used antimicrotubule drug vincristine inhibited platelet aggregation induced by collagen, but not by thrombin, AA, and U46619. These findings provide some information regarding vincristine’s influence on human platelet function.

White et al. reported that the antimicrotubule effect of vincristine did not involve its antiaggregatory effect [17]. Thus, we further determined the exact mechanism underlying vincristine-mediated inhibition of platelet activation by using washed platelets. Our data revealed that vincristine exerts higher inhibitory effects on collagen-induced platelet aggregation. Thus, GPVI signaling was investigated in the present study. The data revealed that vincristine inhibited the activation of Syk and PLCγ2, which are the critical downstream signaling molecules of GPVI. PLCγ2 is well known to cause the formation of diacylglycerol and inositol 1,4,5-trisphosphate, which results in PKC activation and calcium release, respectively [22]. In addition, vincristine inhibits Akt activation, which is the downstream signaling molecule of GPVI [23,24]. In addition, vincristine inhibited the activation of MAPKs, consisting of p38 MAPK, ERK, and JNK. Previously, all the major platelet receptors, including GPVI, have been reported to activate MAPK [5]. p38 MAPK, ERK, and JNK activation can stimulate platelet granule release and promote clot retraction and may regulate GPIIbIIIa activation [5,25,26,27]. Moreover, pharmacological inhibition or gene deletion of MAPKs caused significantly prolonged thrombus formation [5]. Taken together, the results indicate that vincristine may block GPVI signaling, followed by the inhibition of platelet activation events, including granule release and calcium mobilization, eventually suppressing collagen-mediated platelet activation and aggregation.

Integrin α2β1 (GPIa/IIa) is another collagen receptor. Previously, the defective collagen-induced platelet aggregation in the GPIa-deficient platelets was found in patients, which implied that integrin α2β1 may be an essential collagen [28,29]. Moreover, the in vitro studies showed conflicting results. Some studies showed that α2β1 inhibition reduced platelet adhesion in stasis and flow [30,31], while others showed only minor effects [32,33]. These discrepancies are partly due to collagen preparations (soluble and insoluble collagen). Now, integrin α2β1 has been suggested to play a significant but not essential role for the adhesion to collagen [6,34]. Moreover, Nieswandt et al. has reported that GPVI but not integrin α2β1 is essential for platelet interaction with collagen [8]. However, whether vincristine can interfere with integrin α2β1 needs to be further clarified.

Our results from the present study are different from those of previous studies. Steinherz et al. reported platelet dysfunction in vincristine-treated patients whose platelets showed deficiency of secondary aggregation in response to various agonists, except collagen [16]. However, our present study and another study [17] showed that vincristine can inhibit collagen-induced platelet aggregation conducted using platelets from healthy subjects. This discrepancy may be associated with the physical condition of patients with ALL and healthy donors. Similarly, this phenomenon was also observed in dogs. Mackin et al. reported that vincristine has no significant effects on platelet aggregation in response to collagen in clinically normal dogs [20], but Grau-Bassas reported that vincristine can impair collagen-induced platelet aggregation in dogs with lymphoma [19].

Vincristine has dose-limiting neurotoxicity that causes peripheral, symmetric, mixed sensorimotor, and autonomic polyneuropathy [9] because of its large volume of distribution that may limit its efficacy due to the reduction of its exposure or delivery to target tissues [35]. However, hematological toxicity is rare [9]. Although the present study showed that vincristine exerts higher inhibitory effects on collagen-mediated platelet activation, the inhibition of GPVI signaling has been reported to rarely cause bleeding in patients and mice with GPVI deficiency [36]. Moreover, we demonstrated no significant bleeding, as detected by the in vitro PFA-100 assay and the in vivo tail bleeding assay. These findings suggest that vincristine has no tendency to cause bleeding.

Thrombocytosis was observed in vincristine-treated patients with malignancies [37]. This phenomenon of increasing platelet counts was also observed in normal dogs or dogs with lymphoma with clinical vincristine treatment [12,18,20,38]. This ability of vincristine to increase platelet counts has led vincristine or vincristine-loaded platelets to be used to treat patients with ITP [11,12,13,15]. Similarly, vincristine or vincristine-loaded platelets were also used to treat dogs with ITP [39,40]. These observations suggest that, in addition to treating patients with malignancies, vincristine can be used to treat patients with ITP. Moreover, antiplatelet agents were reported to be beneficial in thrombotic thrombocytopenic purpura [21]. Bobbio-Pallavicini et al. reported that antiplatelet agents are useful in treating acute-phase TTP patients or in preventing TTP relapses [21]. These observations suggest that in addition to thrombocytosis, the ability to inhibit platelet activation of vincristine may offer an additional benefit in treating TTP. In addition, TTP is specifically related to a severed efficiency in ADAMTS13 (a disintegrin and metalloproteinase with thrombospondin motifs 13) [41,42,43]. Moreover, dendritic cells (DCs) were also reported to process ADAMTS13 and present its peptides on MHC-II molecules, eventually resulting in the production of ADAMTS13-specific autoantibodies [44]. However, vincristine was reported to induce an immediate increase in platelet count and ADAMTS-13 activity in patients with TTP [45] and exert immunosuppressive effects on DCs [46]. These effects of vincristine on ADAMTS13 and DCs may also contribute to its benefits in treating TTP. On the other hand, it has been reported that COVID-19 adenoviral vector vaccine-induced immune thrombotic thrombocytopenia (VITT) resembles heparin-induced thrombocytopenia, which is mediated by VITT antibodies that can cause platelet activation and subsequent thrombosis through FcγRIIa [47,48,49,50]. However, whether vincristine can exert benefits in post-COVID-19 VITT remains to be investigated.

In conclusion, this study verified the possible mechanisms of the clinically used antimicrotubule agent vincristine for platelet function. We found that vincristine exerts antiplatelet effects at least in part through the suppression of GPVI signaling. Moreover, this property of antiplatelet activity of vincristine may provide its additional benefits in the treatment of TTP.

## 4. Materials and Methods

### 4.1. Materials

Vincristine was purchased from Cayman Chemical (Ann Arbor, MI, USA). Thrombin, arachidonic acid (AA), collagen, and U46619 were purchased from Chrono-Log (Havertown, PA, USA). Fluorescein isothiocyanate (FITC)-conjugated anti–P-selectin antibody was purchased from BioLegend (San Diego, CA, USA). Luciferase/luciferin was purchased from Sigma-Aldrich (St. Louis, MO, USA). Fura 2 acetoxymethyl ester (Fura 2-AM) was purchased from Molecular Probes (Eugene, OR, USA). Anti-Syk, anti–phospho-Syk (Tyr^525/526^), anti–phospho-PLCγ2 (Tyr^759^), anti-PLCγ2, anti–phospho-(Ser) PKC substrate, anti–phospho-p38 MAPK (Ser^180^/Tyr^182^), anti–c-Jun N-terminal kinase (JNK), and anti–phospho-Akt (Ser^473^) polyclonal antibodies and anti-p38 MAPK, anti-p44/42 MAPK, anti–phospho-JNK (Thr^183^/Tyr^185^), and anti-Akt monoclonal antibodies were purchased from Cell Signaling Technology (Danvers, MA, USA). Anti–phospho-p44/42 MAPK (extracellular signal-regulated kinases 1/2 [ERK1/2]; Thr^202^/Tyr^204^) and pleckstrin (p47) polyclonal antibodies were purchased from GeneTex (Irvine, CA, USA). Hybond P polyvinylidene difluoride membrane was purchased from GE Healthcare Life Sciences (Buckinghamshire, UK). Horseradish peroxidase (HRP)-conjugated AffiniPure goat antirabbit, AffiniPure goat antimouse, and AffiniPure donkey antigoat immunoglobulin G (IgG) were purchased from Jackson ImmunoResearch (West Grove, PA, USA). The SuperLight Chemiluminescent HRP Kit was purchased from Bionovas (Toronto, ON, Canada). Vincristine was dissolved in dimethyl sulfoxide (DMSO) and stored at 4 °C until use. Collagen/epinephrine (CEPI) and collagen/ADP (CADP) cartridges were purchased from Dade Behring, Inc. (Marburg, Germany).

### 4.2. Platelet Aggregation

This study was approved by the Institutional Review Board of Taipei Medical University-Joint Institutional Review Board (TMU-JIRB-No. N202104080) and conformed to the principles outlined in the Declaration of Helsinki. All volunteers provided informed consent. Human platelet suspensions were prepared as previously described [51]. In brief, blood was collected from healthy volunteers who had taken no medicine during the preceding 2 weeks and was mixed with an acid-citrate-dextrose (A.C.D.; 56 mM sodium citrate, 65 mM citric acid and 14 mM glucose) solution (9:1 *v*/*v*). Blood samples were allowed to rest at room temperature for 15 min. After centrifugation (120× *g* for 10 min), the supernatant (platelet-rich plasma, PRP) was supplemented with prostaglandin E1 (0.5 μM) and heparin (6.4 IU/mL). After further centrifugation at 500× *g* for 10 min, platelet pellets were washed twice. Washed platelets were suspended in Tyrode’s solution (pH 7.3; 137 mM NaCl, 2.4 mM KCl, 1 mM MgCl_2_, 0.2 mM Na_2_HPO_4_, 12 mM NaHCO_3_, 5.5 mM glucose) containing bovine serum albumin (BSA) (3.5 mg/mL). The final concentration of Ca^2+^ in Tyrode’s solution was 1 mM.

A turbidimetric method was applied to measure platelet aggregation [51] by using a Lumi-Aggregometer (Payton, Scarborough, ON, Canada). Platelet suspensions (3.6 × 10^8^ cells/mL) were preincubated with various concentrations of vincristine (0–75 μM) or an isovolumetric solvent control (0.1% DMSO final concentration) for 3 min before the addition of agonists. The reaction was allowed to proceed for 6 min.

### 4.3. ATP Release and Calcium Mobilization

This method was applied as described previously [52]. In brief, luciferase/luciferin and Fura 2-AM were used to detect ATP release and calcium mobilization, respectively. 3.6 × 10^8^ platelets/mL were preincubated with luciferase/luciferin or Fura 2-AM, and then with vincristine (40 and 75 µM) or 0.1% DMSO for 3 min at 37 °C prior to collagen administration. The intensity of luminescence (ATP release) and the ratio (340/380 nm) of fluorescence (calcium mobilization) were measured using the F-7000 fluorescence spectrometer (Hitachi, Tokyo, Japan) in accordance with the manufacturer’s instructions.

### 4.4. Flow Cytometry

The flow cytometry method was applied as described previously [53]. In brief, 3.6 × 10^8^ platelets/mL were preincubated with vincristine (40 and 75 µM) or 0.1% DMSO; subsequently, collagen was added for 20 min at 37 °C. After the reaction, platelets were fixed with 1% paraformaldehyde for 1 h at 4 °C, washed, and labeled with FITC–conjugated P-selectin antibodies for 30 min to detect the surface expression of P-selectin. After centrifugation and washing, platelets were suspended with 1 mL of phosphate-buffered saline and measured using a flow cytometer (FACScan system; BD Biosciences, San Jose, CA, USA). In the flow cytometry setting, platelets were gated based on the forward scatter and side scatter, and the number of events at 10,000 counts was stopped. All the experiments were performed at least three times to ensure reliability.

### 4.5. Immunoblotting Study

This method was performed as described previously [53]. In brief, platelet suspensions (1.2 × 10^9^ cells/mL) were pretreated with vincristine (40 and 75 μM) or 0.1% DMSO for 3 min and then treated with collagen for 6 min to trigger platelet activation. The reaction was stopped, and the platelets were immediately resuspended in 200 μL of a lysis buffer for 1 h. Lysates were centrifuged at 5000× *g* for 5 min. Samples containing 80 μg of protein were separated through 12% sodium dodecyl sulfate–polyacrylamide gel electrophoresis; proteins were electrotransferred through semidry transfer (Bio-Rad Laboratories, Hercules, CA, USA). Blots were blocked with TBST (10 mM Tris-base, 100 mM NaCl, and 0.01% Tween 20) containing 5% BSA for 1 h and then probed with various primary antibodies. Membranes were incubated with the HRP-conjugated antimouse IgG or antirabbit IgG (diluted 1:3000 in TBST) for 1 h. Immunoreactive bands were detected using an enhanced chemiluminescence (ECL) system. The immunoreactive bands were developed by an ECL kit and analyzed by Celvin S (Biostep, Burkhardtsdorf, Germany). The bands were quantified by ImageJ 1.48v software (National Institute of Health, Bethesda, MD, USA).

### 4.6. Animals

ICR mice (20–25 g, male, 5–6 weeks old) were obtained from BioLASCO (Taipei, Taiwan). All procedures were performed following the Animal Use Protocol of Taipei Medical University (LAC-2021-0158) and were in accordance with the National Institutes of Health Guide for the Care and Use of Laboratory Animals (Eighth Edition, 2011).

### 4.7. Platelet Plug Formation

The Dade Behring platelet function analyzer-100 (PFA-100) system (Marburg, Germany) was used to measure platelet function [54]. Cartridges containing a membrane coated with collagen/epinephrine (CEPI) and collagen/ADP (CADP) were preincubated with normal saline for 2 min. Human whole blood was preincubated with vincristine (40 or 75 μM) or 0.1% DMSO for 3 min. Subsequently, aliquots of human whole blood (0.8 mL/cartridge) were applied to the cartridges before the contents were exposed to high-shear-flow conditions (5000–6000 s^−1^). The closure time was defined as the time required for the platelet plug to occlude the aperture in the membrane.

### 4.8. Tail Bleeding Assay

Mice were anesthetized with a mixture containing 75% air and 3% isoflurane and were maintained in 25% oxygen, and they were intravenously administered DMSO (solvent control) and vincristine (36 or 68 mg/kg) or aspirin (20 mg/kg, positive control) for 10 min. Immediately, bleeding was induced by severing the tail 3 mm from the tail tip, and the bleeding tail stump was immersed in saline. Subsequently, the bleeding time was continually recorded until no sign of bleeding was observed for at least 10 s. The dose for mice was converted from the dose for humans [55].

### 4.9. Data Analysis

The experimental results are expressed as mean ± standard error of the mean and are accompanied by the number of observations (*n*). Values of *n* refer to the number of experiments, each of which was conducted with different blood donors. All experimental results were assessed using analysis of variance (ANOVA). If ANOVA indicated significant differences in the group means, each group was compared using the Newman–Keuls method. *P* < 0.05 was considered statistically significant.

## Data Availability

All data generated or analyzed in this study are included within this article.

## Sample Availability

Samples of the compounds are not available from the authors.
